# Cutaneous sporotrichoid leishmaniasis: An atypical case caused by *Leishmania major*

**DOI:** 10.1016/j.idcr.2022.e01629

**Published:** 2022-11-01

**Authors:** Adila Bassaid, Yassine Merad, Mohand Ouali Ait Si Ali, Assya Djeridane, Fatma Bachi, Haiet Adjmi-Hamoudi

**Affiliations:** a Service de Parasitologie-Mycologie, CHU Mustapha, Algeria; b Laboratoire Central, Parasitologie-mycologie, Hopital “Hassani Abdelkader”, Algeria; c Service de dermatologie HCA, Algeria; d Service de biologie parasitaire IPA Dely Brahim, Algeria; e Service de Parasitologie-mycologie HCA, Algeria

**Keywords:** Sporotrichoid leishmaniasis, Cutaneous leishmaniasis, Leishmania major, Vectorborne disease, Rare leishmaniasis

## Abstract

A 32 year-old male was referred in our institution for painless erythematous papules on the back of the right hand, treated by various local therapies with no noticeable benefits. On examination multiples inconspicuous nodules were identified 10 days after the onset of the primary lesion. Mycological examination was done to rule out sporotrichosis, then cutaneous leishmaniasis was retained by finding amastigotes forms, and *Leishmania major* agent was confirmed after culture on NNN medium followed by isoenzyme electrophoresis. Sporortrichoid leishmaniasis is a rare condition and usually due to extension of local leishmaniasis into the subcutaneous tissue via direct extension, bloodstream or lymphatics. The patient responded favorably to Meglumine antimoniate treatment. To our knowledge, sporotrichoid cutaneous leishmaniasis after an erythematous-papular onset has never been reported in Algeria, this clinical entity should be considered for an earlier diagnosis and specific therapy.

## Introduction

Leishmaniasis is a parasitic vector-borne disease, inoculation occurs after a Sandfly bite. The three major clinical types are cutaneous leishmaniasis (CL), mucosal leishmaniasis (ML), and visceral leishmaniasis (VL) [1].

The lesions usually appear in non-covered regions of the body [2]. CL has a broad spectrum of clinical manifestations.

Atypical forms of CL include sporotrichoid leishmaniasis (SL), which is usually described as a primary ulcer associated with lymphagitis and nodules [3].

Sporotrichoid cutaneous leishmaniasis is considered as a dissemination of amastigotes via the lymphatics to the subcutaneous tissues [3], this particular clinical entity has been described in Africa and seems to be significantly more reported in Sudan [4].

We describe an unusual case of SL, the disease began as an erythematous-papular rash located on the right hand.

## Case presentation

We report on a 32 year-old male with a history of contact eczema, residing at Djelfa town, in Algeria.

**Fig. 1:**
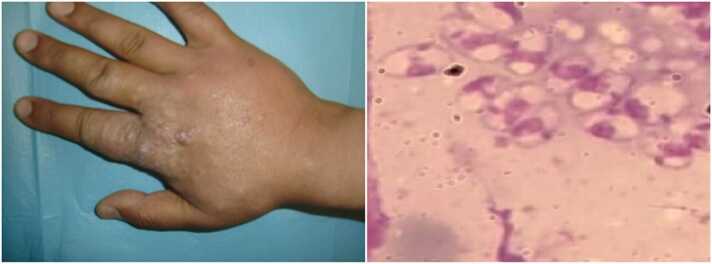
A) Erythematous and papular plaque on the right hand, B) Giemsa stained smear showing Leishmania amastigote forms.

**Fig. 2:**
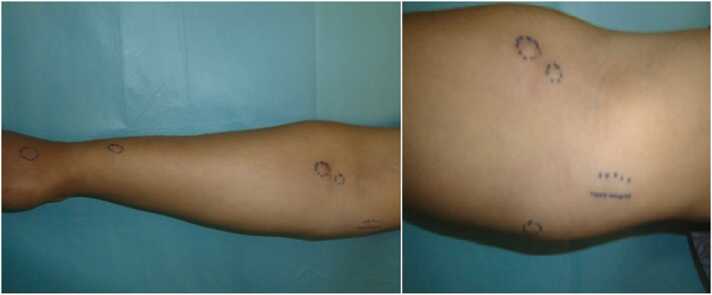
Subcutaneous nodules in the lower and upper third of forearm.

The patient reported a 6-week history of painless, and non-healing skin lesions on his right hand, he admitted having applied topical corticosteroids and antiseptics without any improvement, and then he had received two courses of antibiotics (pristinamycin, amoxicillin + clavulanic acid), with no significant results, moreover, there was an extension of the lesions leading to an imperative reevaluation of the condition. After being referred to our institution, the patient presented limited erythematous-papular plaque of 8 cm of diameter, located on the dorsal side of the right hand, the lesion was formed by the confluence of several papules (Fig. 1), with satellite lesions on the periphery, the initial plaque was associated with several firm and painless subcutaneous nodules 0.5–1.5 in diameter, following a lymphatic drainage to the elbow joint (Fig. 2). The patient didn’t remembered being bitten by an insect, and other body surfaces did not reveal similar lesions. Furthermore examination of the other systems was unremarkable. Parasitological examination revealed abundant amastigote forms of *Leishmania sp* (Fig. 1), while direct mycological examination as well as the culture on Sabouraud medium at 25 °C, and on the BHI medium (Brain Heart Infusion) at 37 °C were both negative for fungal presence. In addition, culture on NNN medium (Novy- MacNeal-Nicolle), associated to species identification based on isoenzyme electrophoresis revealed *Leishmania major* as etiological agent.

**Fig. 3:**
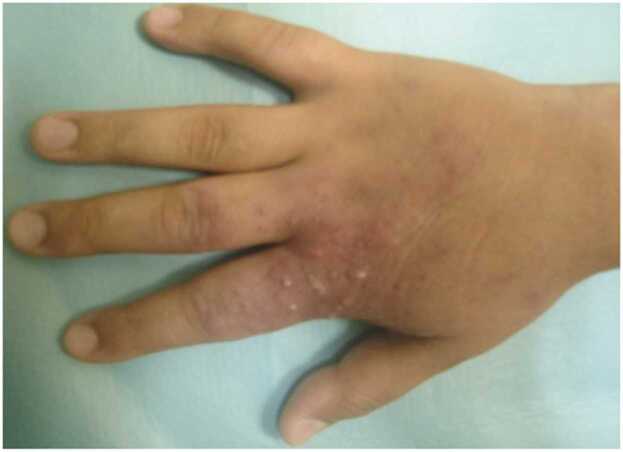
Improvement of lesions after 10 days of Glucantime treatment.

The patient was started on Meglumine antimoniate (Glucantime©) as first line treatment 60 mg/kg/day for 15 days, which induced a reduction of the lesions after 10 days (Fig. 3), and favorable outcome at day 15 of follow-up.

## Discussion

SL is a rare form of Old World cutaneous leishmaniasis, mostly due to *Leishmania aethiopica*, it has been already observed in Africa, among 2059 patients with cutaneous leishmaniasis in Tunisia, 34 (1.7%) developed SL [3], furthermore, SL was significantly reported in Sudan, occurring in 23% of patients compared with 10% in Saudi Arabia [4], [5], whereas this form seems to be less habitual in other *Leishmania major* endemic areas like Pakistan, Morocco and Turkey [6], [7], [8].

Leishmaniasis is associated to environmental changes, such as deforestation, wide urbanization, irrigation and construction of dams [2].

According to gender SL is more commonly described in female than males [3].

Dissemination of CL is not a common phenomenon [8], our SL case seems to be caused by locoregional diffusion via the lymphatic route (Fig), and the species found is *Leishmania major*, which rarely gives sporotrichoid forms.

Often subcutaneous nodules are small, inconspicuous and difficult to visually assess [8]. Although the number of sporotrichoid nodules is reported to be variable, in a majority of patients multiples nodules can trigger spontaneously a few days after the onset of the primary lesions [3], [8], according to a Tunisian study the nodules number can reach 20 [9]. Furthermore, leishmaniasis nodules are mainly reported on forearms and arms, but lower limbs are less affected [3], [10]. The subcutaneous nodules in our case were small, slightly raised, located in the lower and upper third of the forearm, they were also palpable, and had a firm painless consistence. The papules surrounding an ulcerated nodule is a typical feature in favor of CL [8], however our clinical picture was not highly suggestive of SL.

Few diseases can produce a sporotrichoid dissemination of chronic foci, namely infections by *Sporothrix schkenkii*, atypical mycobacteria, *Nocardia brasiliensis* and *Leishmania sp.,* among others [11]. In summary, the list of causative pathogens varies substantially as a function of exposure-prone activities, travel history, geographic location, occupation and hobbies. The main characteristics of the most common diseases are depicted in Table 1.

**Table 1:** Characteristics of the main sporotrichoid lesions.

Pathogenic Agent	Contamination	Clinical particularities	Treatment
*Leishmania sp*	Sandfly bite after travelling or living in an endemic area	Papular or ulcerative lesions on uncovered regions of body	Meglumine antimoine 20 kg/kg daily for 20 days
*Mycobacterium marinum*	Contact with water from aquarium, fish-handling, swimming in lakes, pools or ocean	Papulo-nodules evolving slowly, mainly on upper limb Rarely joints and bone infections	Rifampin 5 mg/kg daily For several months
*Nocardia sp*	Gardening, soil contamination, splinters.	Can affect skin, lung, brain, mainly in immunosuppressed individuals.	Bactrim 2 tablets three times daily during 3 months
*Sporothrix schenckii*	Minor trauma by contaminated plant matter, animal scratches or bites	Papulo-nodules involving hands or arms Dissemination if immunosuppression	Itraconazole 200 mg daily, during two months

Sporotrichoid dissemination of cutaneous leishmaniasis can appear after local traumatism or puncture diagnostic [11], however no local trauma was reported by our patient, nevertheless we recorded local application of corticosteroids, which probably can lead to the dissemination by a reduction in local immunity. Besides, it has been also suggested that tissue damage provoked by local therapy triggers the spread of leishmaniasis into the subcutis and lymphatic ducts [12]. Furthermore, the patient’s residence could contain strains of varying virulence that are irregularly distributed across transmission foci [3].

Culture of the scraping samples on NNN medium is not always necessary for diagnosis, but it could be contributive in pauceparasitic forms, and should be done for species typing purpose.

Molecular identification and isoenzymes electrophoresis typing have revealed that sporotrichoid leishmaniasis has largely been associated with *Leishmania major* species [3], [10], [13].

Althougt sporotrichoid cutaneous leishmaniasis is uncommon in Algeria, similar presentations mainly include sporotrichosis that has already been reported in the country.

According to literature, Treatment with intramuscular 20 mg/Kg/day meglumine antimoniate (Glucantime©) for 15 days, is effective [8].

## Conclusion

Sprotrichoid leishmaniasis is a rare condition, diagnosis should be guided by the patient history, physical examination and laboratory examination, especially in areas with known predominance of *Leishmania major*.

## Funding statement

Authors declare no fundings.

## CRediT authorship contribution statement

Adila Bassaid, Yassine Merad, Mohand Ouali Ait Si Ali, Assya Djeridane^,^ Fatma Bachi, Haiet Adjmi-Hamoudi helped equally to collect data, writing,and reviewing.

## Conflicts of interest

Authors declare no conflicts of interests.

## Data Availability

All data is available in this paper
